# Veterinary Medical Society of Harvard College, Class of ’99

**Published:** 1897-05

**Authors:** C. Bowley

**Affiliations:** Secretary


### VETERINARY MEDICAL SOCIETY OF HARVARD COLLEGE, CLASS OF '99.

THE regular monthly meeting was called to order by President Kennelly, of Springfield, Mass., March 25, 1897. Many subjects of importance were discussed, among which was an original essay by J. Francis Conners, on the "Luxations of the Patella."

New officers were elected as follows: President, J. Francis Conners, South Boston, Mass.; Vice-President, T. J. Coyne, Charlestown, Mass.; Treasurer, Willis A. J. Dillingham, Berwick, Maine; Secretary, J. E. Kennelly, Springfield, Mass. Board of Directors: Daniel P. O'Brien, Chairman, Chelsea, Mass.; Clifton Bowley, Lynn, Mass.; W. Lowering, Milford, N. H.; and N. C. Koons, Roxbury, Mass.

Mr. Dinsmore and Mr. Wadsworth were appointed to wait on the dean in reference to certain original investigations.

The Class of '99 has representatives of all the New England States. The regular monthly meetings will take place on the second Monday of each month.

C. Bowley,
Secretary.

